# Targeting Mutated Plus Germline Epitopes Confers Pre-clinical Efficacy of an Instantly Formulated Cancer Nano-Vaccine

**DOI:** 10.3389/fimmu.2019.01015

**Published:** 2019-05-15

**Authors:** Mona O. Mohsen, Monique Vogel, Carsten Riether, Julius Muller, Silvia Salatino, Nicola Ternette, Ariane C. Gomes, Gustavo Cabral-Miranda, Aadil El-Turabi, Christiane Ruedl, Thomas M. Kundig, Said Dermime, Alexander Knuth, Daniel E. Speiser, Martin F. Bachmann

**Affiliations:** ^1^Nuffield Department of Medicine, Jenner Institute, University of Oxford, Oxford, United Kingdom; ^2^Department of BioMedical Research, Immunology RIA, University Hospital of Bern, Bern, Switzerland; ^3^National Center for Cancer Care & Research, Doha, Qatar; ^4^Department of Medical Oncology, University Hospital of Bern, Bern, Switzerland; ^5^Wellcome Centre for Human Genetics, University of Oxford, Oxford, United Kingdom; ^6^Division of Molecular Genetics and Cell Biology, Nanyang Technological University, Singapore, Singapore; ^7^Department of Dermatology, University of Zurich, Zurich, Switzerland; ^8^Department of Oncology, University of Lausanne, Lausanne, Switzerland

**Keywords:** virus-like particles, vaccine, personalized, melanoma, neoantigen, mutated, germline

## Abstract

Personalized cancer vaccines hold promises for future cancer therapy. Targeting neoantigens is perceived as more beneficial compared to germline, non-mutated antigens. However, it is a practical challenge to identify and vaccinate patients with neoantigens. Here we asked whether two neoantigens are sufficient, and whether the addition of germline antigens would enhance the therapeutic efficacy. We developed and used a personalized cancer nano-vaccine platform based on virus-like particles loaded with toll-like receptor ligands. We generated three sets of multi-target vaccines (MTV) to immunize against the aggressive B16F10 murine melanoma: one set based on germline epitopes (GL-MTV) identified by immunopeptidomics, another set based on mutated epitopes (Mutated-MTV) predicted by whole exome sequencing and a last set combines both germline and mutated epitopes (Mix-MTV). Our results demonstrate that both germline and mutated epitopes induced protection but the best therapeutic effect was achieved with the combination of both. Our platform is based on Cu-free click chemistry used for peptide-VLP coupling, thus enabling bedside production of a personalized cancer vaccine, ready for clinical translation.

## Introduction

Melanoma is the most aggressive type of skin cancer mostly due to its high metastatic potentials (1). The complexity of melanoma phenotype affects its responsiveness to various therapies. Therefore, scientists emphasize the need for personalized treatments, including vaccinations that target neoantigens representing the uniqueness of each patient's tumor (2, 3). Neoantigens are considered attractive because T-cells specific for these altered-self antigens are not expected to be attenuated by immune tolerance mechanisms as compared to T-cells specific for conserved self-antigens (4–6). Given the facts that powerful tumor infiltrating T-cells recognize multiple tumor antigens (7), and that targeting of a single antigen allows tumors to relapse (8), it is desirable to target multiple antigens (3, 9, 10). However, identifying sufficient numbers of neoantigens may be difficult for many human tumors that often show low mutational burden such as prostate and pancreatic cancers (11–13). Targeting neoantigens by using synthetic long peptides (14), RNA-based vaccines (15), or DC-based vaccines (16) have so far only been done in patients with tumors of high mutational burden, essentially melanoma.

We have shown previously that VLPs derived from the bacteriophage Qβ loaded with A-type CpGs and coupled to MelanA/MART-1 peptide can be efficiently processed by murine and human DCs for MHC-I presentation and induced strong CD8^+^ T-cell responses in HLA-A2 transgenic mice as well as in melanoma patients (17, 18). Recent research has also shown that CMP-001, a product that is also based on Qβ-VLPs loaded with the same A-type CpGs induced tumor regressions in advanced melanoma patients treated by direct intra-tumoral injection, even though the VLPs used were not linked to tumor-specific antigens. Three clinical trials for CMP-001 are currently ongoing (NCT02680184, NCT03084640, and NCT03618641).

In this study, we have utilized VLPs to build up a personalized multi-target vaccine based on identified germline and predicted mutated peptides coupled to TLR 9-ligand loaded VLPs by the non-toxic bio-orthogonal Cu-free click chemistry. The generated vaccine candidates induced effective CD8^+^ T-cell responses and the strategy can also be implemented for solid tumors with low mutational burdens as identification of germline epitopes with high affinity to MHC-I molecules is always feasible.

## Materials and Methods

### Production and Purification of Qβ-VLPs

Qβ-VLPs expression and production was performed as previously described in detail in Kozlovska et al. (19), Cielens et al. (20), and Storni et al. (21).

### Packaging Qβ-VLPs With B-Type CpGs

B-type CpGs (5″-TCC ATG ACG TTC CTG ATG CT-3″) with phosphorothioate backbone was custome made by (IBA). Twenty mM HEPES (Sigma Aldrich) was used to solubilize Qβ-VLPs and RNase A (Merck) was used to digest the naturally packaged RNA inside Qβ-VLPs (1.2 mg/ml RNaseA/ 3 mg/ml Qβ-VLPs) for 3 h at 37°C. The digestion was confirmed using 1% agarose gel and SYBR safe dye. Qβ-VLPs was then repackaged with B-type CpGs (1.125 μg/ 20 μg Qβ-VLPs) and packaging was also confirmed by using 1% agarose gel stained by SYBRsafe stain, while Qβ-VLPs protein was detected by subsequent staining with coomassie blue.

### Generation of Qβ(CpGs)-p33 Vaccine Using SMPH Chemistry

B-type CpGs were packaged in Qβ-VLPs as described earlier and then derivatized for 1 h at RT using SMPH (Succinimidyl 6-((beta-maleimidopropionamido)hexanoate)) (Thermo Fisher Scientific). Excess SMPH cross-linker was removed using Amicon centrifuge tubes of 100 kDa MWCO (Sigma-Aldrich). Modified p33 peptide—derived from Lymphocytic Choriomeningitis Virus LCMV—was synthesized by the addition of GGC amino acids (a.a.) at the C-terminus “H-KAVYNFATMGGC-NH2” (Pepscan BRESTO) to facilitate its coupling to SMPH cross-linker. The modified peptide was reconstituted in DMSO and added in 10-fold molar excess over Qβ(CpGs)-VLPs monomer. The mixture was incubated for 1 h at RT and excess peptide was removed using Amicon centrifuge tubes. The coupling efficiency was tested by SDS-PAGE stained with coomassie blue and assessed by densitometric analysis of the SDS-PAGE by comparing Qβ-VLP monomer bands to Qβ-VLP monomer plus p33 after coupling.

### Generation of Qβ(CpGs)-p33 Vaccine and Multi-Target Vaccines Using DBCO (Cu-Free Click Chemistry)

B-type CpGs were packaged in Qβ-VLPs as described earlier and then derivatized for 30 min at RT using DBCO (dibenzocyclooctyne NHS ester) (Sigma-Aldrich). Excess DBCO cross-linker was removed using Amicon centrifuge tubes of 100 kDa MWCO (Sigma-Aldrich). Modified p33 peptide was synthesized by the addition of GGCK a.a. and an azide (N3) group at the C-terminus “H-KAVYNFATMGGCK(N3)-NH2” (Pepscan BRESTO) to facilitate its coupling to DBCO cross-linker. The peptides for the multi-target vaccines (MTV) were synthesized by the addition of extra 4 a.a. using their flanking protein sequence and an azide (N3) group at the C-terminus. The generation of multi-target vaccine was done for each peptide separately. The modified peptides were reconstituted in DMSO and added in 10-fold molar excess over Qβ(CpGs)-VLPs monomer. The vaccine was incubated for 30 min at RT and excess peptide was removed using Amicon centrifuge tubes. The efficiency of the coupling was tested as explained in section Generation of Qβ(CpGs)-p33 vaccine using SMPH chemistry.

### Mice

Wild type C57BL/6 mice were purchased from Harlan. *Rag2*^−/−^ mice on a C57BL/6 background were provided by Ochsenbein' lab and bred in our pathogen-free animal facility. All *in vivo* experiments used 8–12-week-old female. All animal procedures were performed in accordance with the Swiss Animals Act (455.109.1) (September 2008, 5th) of University of Bern.

### Measuring p33 Specific T-Cell Response With Tetramers

P33 (H-KAVYNFATM-NH2) tetramers designed with H2-D^b^ allele and APC or PE fluorochrome (TCMetrix) was used to measure p33 specific T-cells. WT C57BL/6 mice (8–12 weeks old; Harlan) were vaccinated s.c. once with 50 μg Qβ(CpGs)-p33 vaccine coupled with SMPH or DBCO cross-linkers. Seven days later spleens were collected and smashed using 70 μM cell strainer (Sigma-Aldrich). Cells were washed 1x with sterile PBS and RBCs were lysed using ACK lysis buffer. ~1 × 10^6^ cells were collected in 96-well V-bottom plate and stained with p33 tetramers (TCMetrix) followed by anti-CD8α (53–6.7, BD Biosciences) for flow cytometric analysis by FACSCanto and FlowJo Software.

### Intra-cellular Cytokine Staining for IFN-γ

Intra-cellular cytokine staining was performed on spleens or TILs of vaccinated mice to measure IFN-γ. ~2 × 10^6^ cells were collected from spleen or TIL and pulsed with 1 μg of p33 peptide or with the mixture of germline peptides or mutated peptides or both according to the vaccine group for 6 h at 37°C with 1:1,000 Brefeldin A and Monensin (BD Biosciences). Cells were collected and washed 3x with sterile PBS/0.1% BSA. ~1 × 10^6^ cells were collected in 96-well V-bottom plate and stained with anti-CD8α (53–6.7, BD Biosciences). Cells were then fixed with 100 μl of the fixation buffer (Thermo Fisher Scientific) and permeabilized with 1x of the permeabilization buffer (Thermo Fisher Scientific). Cells were stained with anti-IFN-γ (XMG1.2, BD Biosciences) for flow cytometric analysis by FACSCanto and FlowJo Software.

### CFSE *in vivo* Cytotoxic Assay

WT C57BL/6 mice (8–12 weeks old; Harlan) were vaccinated with a single s.c. injection of 50 μg Qβ(CpGs)-p33 vaccine coupled with SMPH cross-linker or DBCO cross-linker. Seven days later, target splenocytes from naïve WT C57BL/6 mice were collected and RBCs were lysed and divided into 2 groups. The 1st naïve splenocyte group was labeled with 2 μM CFSE (Thermo Fisher Scientific) and kept un-pulsed while the 2nd naïve splenocytes group was first pulsed with 1 μg p33 and then labeled with 10 μM CFSE. The prepared target groups were mixed in 1:1 ratio and each previously vaccinated mouse received 1 × 10^7^ of un-pulsed CFSE^Low^ cells and 1 × 10^7^ of pulsed CFSE^Hi^ cells intravenously. Four hours later the spleens of the vaccinated mice were collected and analyzed by flow cytometry for frequency of CFSE^Low^ and CFSE^Hi^. Specific lysis for each group was measured using the formula “Ratio = 100X(1–CFSE^Hi^ pulsed/CSFE^Low^ un-pulsed).”

### Immunopeptidomics

B16F10 melanoma cell line transfected with p33 peptide was provided by Ochsenbein's lab and cultured in T-150 cm^2^ flask using Dulbecco's modified Eagle's medium (DMEM) with 10%FBS and 1% Streptomycin/Penicillin. Cells at 80% confluency were washed 2x with sterile PBS and collected with scrapers, centrifuged and freezed at −80°C. Cells were lysed using 5 ml lysis buffer (1% Igepal, 300 mM sodium chloride, 100 mM Tris, pH 8.0) supplemented with protease inhibitor cocktail (Roche) for 30 min on ice. Lysates were cleared by two centrifugation steps at 500 g for 10 min and then 20,000 g for 60 min. One mg of anti-mouse MHC class I antibody (ATCC HB-51) was bound and cross-linked to 1 ml Protein G beads (GE healthcare). Lysates were incubated with the antibody beads over night at 4°C and washed subsequently with 50 mM Tris, pH 8.0 containing either 150, 450 mM, and no salt. Peptides were eluted with 5 ml 10% acetic acid and concentrated in a vacuum concentrator (Eppendorf). Peptides were then injected onto a 4.6 × 50 mm ProSwift RP-1S column on an Ultimate 3000 system (Thermo Fisher Scientific). Peptides were separated from larger complex components by elution using a 500 μl/ min flow rate over 10 min from 2 to 30% ACN in 0.1% TFA. Alternate fractions that did not contain the beta-2-microglobulin were pooled and two final fractions were analyzed by nano-ultra performance liquid chromatography tandem mass spectrometry (nUPLC-MS^2^) on a Fusion Lumos (Thermo Fisher Scientific). Peptides were separated on a Ultimate 3000 RSLCnano system (Thermo Fisher Scientific) using a PepMap C18 column, 2 μm particle size, 75 μm × 50 cm (Thermo Fisher Scientific) with a 1 h linear gradient of 3–25% buffer B (0.1% formic acid, 5% DMSO in acetonitrile) in buffer A (0.1% formic acid, 5% DMSO in water) at a flow rate of 250 μl/ min. Peptides were introduced using an Easy-Spray source at 2000 V to a Fusion Lumos (Thermo Fisher Scientific) at 305°C. Full MS spectra were recorded from 300 to 1,500 m/z in the Orbitrap at 120,000 resolution with an AGC target of 400,000. Precursor selection was performed using TopSpeed mode at a cycle time of 2 s. Peptide ions were isolated using an isolation width of 1.2 amu and trapped at a maximal injection time of 120 ms with an AGC target of 300,000. Higher-energy collisional dissociation (HCD) fragmentation was induced at an energy setting of 28 for peptides with a charge state of 2–4, while singly charged peptides were fragmented at an energy setting of 32 at lower priority. Fragments were analyzed in the Orbitrap at 30,000 resolution. Analysis of raw data was performed using Peaks 7.5 software (Bioinformatics Solutions). Sequence interpretation of MS^2^ spectra was carried out using databases containing all mus musculus UniProt database entries at a FDR of 1.4. Motif analysis of common a.a. in peptide sequences was performed using WebLogo 3.5 (weblogo.threeplusone.com). Peptide binding predictions were performed using NetMHCpan 4.0.

### Whole Exome Sequencing

B16F10 melanoma cell line was cultured and collected as described earlier. DNA was isolated using Purelink genomic DNA mini kit according to manufacturer's instructions (Thermo Fisher Scientific), and gave a yield of 394 ng/ul. The quality of the DNA yield was assessed by loading an aliquot on 2% agarose gel. A library was prepared using Agilent SureSelect XT, based on the cRNA-baits targeted capture. The Mouse All Exon kit captures 49.6 Mb region that covers 221,784 exons within 24,306 genes. Sequencing was performed using HiSeq 2,500 machine with paired end 2x 25 run, aiming × 100 (raw data) exome coverage by FASTERIS.

### Bioinformatics Analysis of Whole Exome Sequencing

Sequenced reads from the B16F10 melanoma cell line sample were mapped to the Mus musculus reference genome assembly GRCm38 from NCBI using the software BWA-MEM (22) with default parameters. Supplementary alignments were removed and the resulting BAM file was sorted with SAMtools (23). Mapped reads were subsequently used as input for the variant calling software Platypus (version 0.8.1, specifying the option “–minFlank = 0”) (24) to identify SNPs and short indels (<50 bp). The 74138 identified variants were annotated with standard R packages using GENCODE and further investigated with the Variant Effect Predictor Ensembl frame-work (VEP, release 90) (25). Peptides were extracted using a sliding window from +/−8AA to +/−14AA around the mutation. HLA type prediction for H2-D^b^ and H2-K^b^ alleles was performed using NetMHC4.0.

### *In vitro* T-Cell Assay

1 × 10^6^ B16F10 melanoma cells were injected into the flank of C57BL/6 *RAG2*^−/−^ mice (8–12 weeks old). Twelve days later the growing tumors were collected and processed for transplantation into the flank of WT C57BL/6 mice (8–12 weeks old; Harlan) under anesthesia. The tumors were allowed to grow without any treatment for 14 days (set as the humane end point 1,000 mm^3^). Tumors were collected and digested by DNaseI (Boehringer) and collagenase D (Roche) in 5% FSC containing DMEM for 1 h at 37°C. Tumors were then smashed using 100 μm cell strainer (Sigma-Aldrich) and washed 2x with PBS. Tumor-infiltrating lymphocytes (TILs) were separated using Ficoll-paque premium (GE Healthcare) by centrifugation for 30 min at RT at 1,800 rpm. TILs were collected and kept on ice. At the same time bone marrow derived dendritic cells (BMDCs) were prepared from naïve WT C57BL/6 mice (8–12 weeks old; Harlan). Briefly, bone marrow cells were cultured in RPMI supplemented with 10% FCS, glutamine, sodium pyruvate, penicillin, and streptomycin for 6–8d in the presence of granulocyte-macrophage colony-stimulating factor containing X-63 cell supernatant. BMDCs were then pulsed with the selected peptides separately for 1 h at 37°C. The pulsed BMDCs were then washed 3x with DMEM medium and co-cultured with the collected TILs for 6 h at 37°C with (1:1,000) Brefeldin A and Monensin (BD Biosciences). ICS staining was carried out as described in section Intra-cellular cytokine staining for IFN-γ.

### Measuring Treg

Peripheral blood from WT C57BL/6 mice (8–12 weeks old; Harlan) vaccinated with MTV was collected in heparin. RBCs were lysed with ACK lysing buffer (Thermo Fisher Scientific) and cells were stained with anti-CD4 (RM4-5, eBioscience) and anti-CD25 mAb (CD25-4E3, BD Biosciences) for flow cytometric analysis by FACSCanto and FlowJo Software.

### Tumor Experiments and Survival Rate

1 × 10^6^ B16F10 melanoma cells were injected into the flank of C57BL/6 *RAG2*^−/−^ mice (8–12 weeks old). Twelve days later the growing tumors were collected and processed for transplantation into the flank of WT C57BL/6 mice (8–12 weeks old; Harlan). Vaccination was carried 5 days later as described in Table 1.

**Table 1:** The prepared groups, treatment, and route of injection.

**Group**	**Treatment**	**Route of injection**
1	120 μg Qβ(CpGs)-VLPs	s.c.
2	10 μg anti-CD25 (PC61, Biolegend)	i.v.
3	Germline multi-target vaccine (GL-MTV) 120 μg containing: 20 μg Qβ(CpGs)-PMEL17 20 μg Qβ(CpGs)-MTC-1 20 μg Qβ(CpGs)-Calpastatin 20 μg Qβ(CpGs)-ZFP518 20 μg Qβ(CpGs)-TRP-2 20 μg Qβ(CpGs)-Caveolin2	s.c
	10 μg anti-CD25 (PC61, Biolegend)	i.v.
4	Mutated multi-target vaccine (Mutated-MTV) 120 μg containing: 60 μg Qβ(CpGs)-Cpsf3l 60 μg Qβ(CpGs)-Kifl8b	s.c.
	10 μg anti-CD25 (PC61, Biolegend)	i.v.
5	Mix multi-target vaccine (Mix-MTV) 120 μg containing: 15 μg Qβ(CpGs)PMEL17 15 μg Qβ(CpGs) MTC-1 15 μg Qβ(CpGs)-Calpastatin 15 μg Qβ(CpGs)-ZFP518 15 μg Qβ(CpGs)-TRP-2 15 μg Qβ(CpGs)-Caveolin2 15 μg Qβ(CpGs)-Cpsf3l 15 μg Qβ(CpGs)-Kifl8b	s.c.
	10 μg anti-CD25 (PC61, Biolegend)	i.v.

Tumors were collected 14 days later as the control groups reached the humane end-point of 1,000 mm^3^. Tumor volume was measured using a caliper according to the formula V = (W × W × L)/2, where V is tumor volume, L is tumor length and W is tumor width. TILs were isolated as described earlier. TILs were stained with anti-CD8α (53–6.7, BD Biosciences), anti-IFN-γ (XMG1.2, BD Biosciences), anti-CD45 (30-F11, Biolegend), anti-Ly6G (1A8, Biolegend), and anti-Ly6C (HK1.4, Biolegend) for flow cytometric analysis by FACSCanto and FlowJo Software. Survival was evaluated using the same designated vaccines and the transplanted WT C57BL/6 mice (8–12 weeks old; Harlan) were vaccinated twice a week and tumor size was monitored daily and measured using a caliper.

### Statistics

Data have been presented as mean ± SEM using GraphPad PRISM 7.0d. Comparison between the groups was performed by Student's *t*-test or one-way ANOVA. Tumor growth curves were analyzed by AUC and survival was analyzed by Log-rank (Mantel-Cox) test. *P*-values ^****^P < 0.0001; ^***^P < 0.001; ^**^P < 0.01; ^*^P < 0.05.

## Results

### Algorithm for the Generation of a Personalized Melanoma Vaccine Platform Based on VLPs

**Figure 1:**
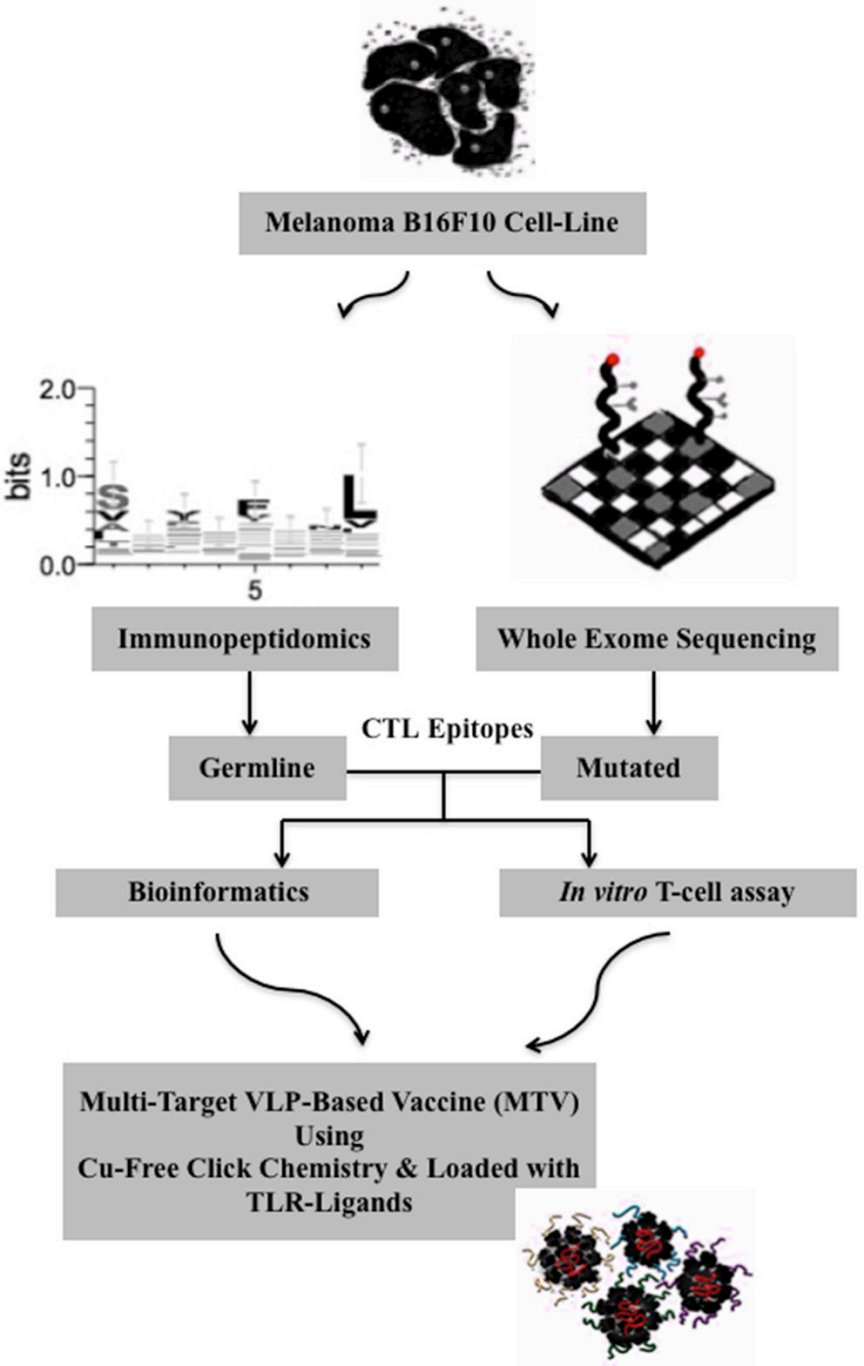
Algorithm for the generation of a personalized melanoma vaccine platform based on VLPs. First, tumor-specific epitopes need to be identified in a systematic way. Here, immunopeptidomics approach has been used to identify potential tumor-specific germline epitopes and whole exome sequencing to predict the mutated ones. In a second step, the identified and predicted epitopes should be prioritized and this was basically achieved by bioinformatics and stimulation of tumor-infiltrating cells using *in vitro* T-cell assay. Next, the selected epitopes are extended to ~13–14 amino acids long peptides using their flanking protein sequence for the goal of targeting CD8^+^ CTL. The extended peptides are then synthesized and coupled to CpG-loaded VLPs using Cu-free click chemistry. A mix-multi target VLP-based vaccine is proposed.

We adapted the Qβ-VLP/CpG platform for the generation of a personalized cancer vaccine in mice as illustrated in Figure 1. Using immunopeptidomics and whole exome sequencing we identified and predicted tumor-specific germline and mutated CTL epitopes of the B16F10 melanoma cell line. The identified and predicted epitopes were then prioritized and validated by bioinformatics and *in vitro* experiments. We used bio-orthogonal copper (Cu)-free click chemistry; a key step in our platform to couple the selected peptides to CpG-loaded Qβ-VLPs to form MTV. The vaccines were produced with germline epitopes, germline-multi target vaccine (GL-MTV) or mutated epitopes (Mutated-MTV) or a combination of both (Mix-MTV) in mice transplanted with aggressive B16F10 melanoma tumors.

### Bio-orthogonal Cu-Free Click Chemistry; an Efficient Method for Coupling Antigens to VLPs to Enhance Their Immunogenicity

**Figure 2:**
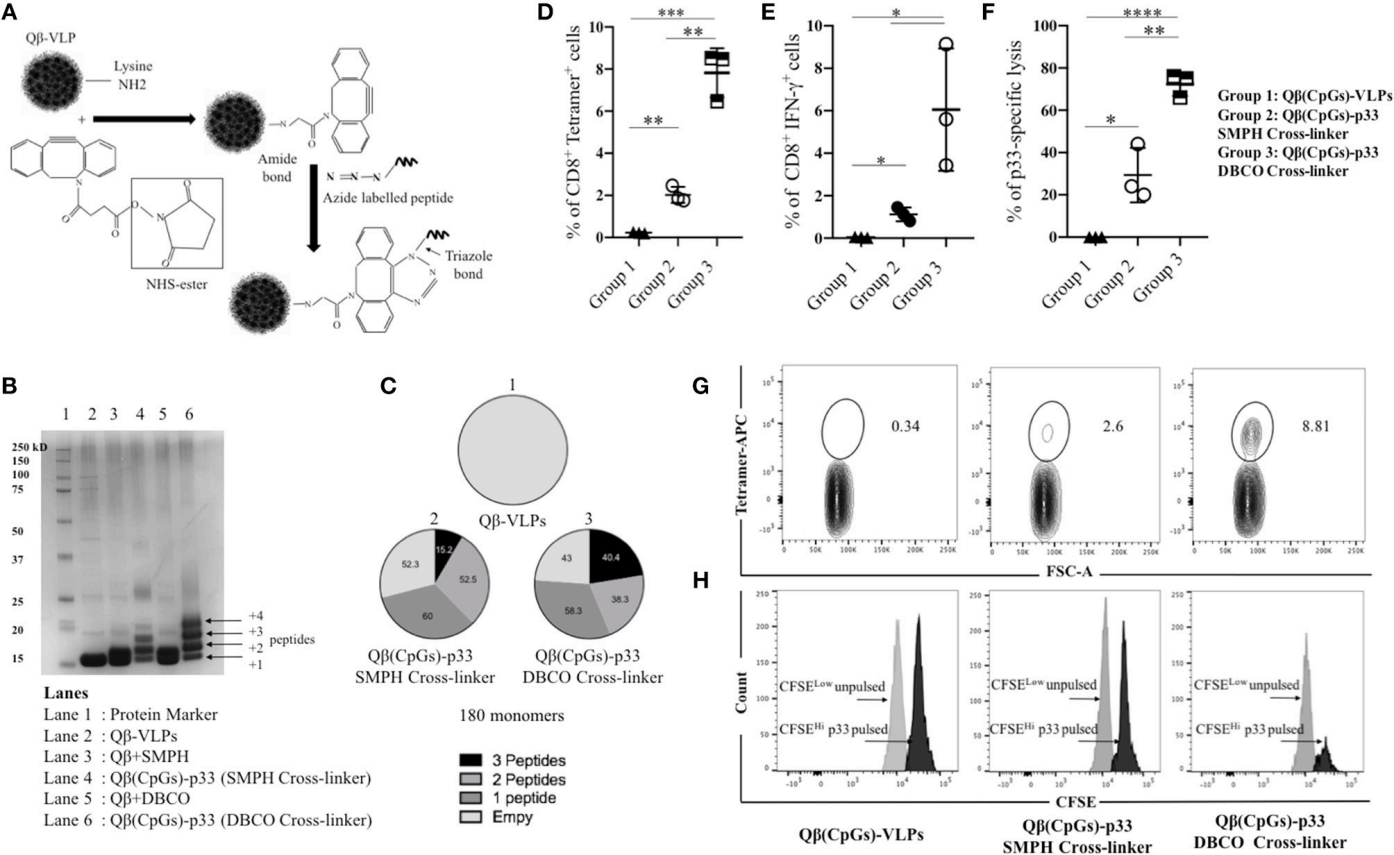
Bio-orthogonal Cu-free click chemistry; an efficient method for coupling antigens to VLPs to enhance their immunogenicity. **(A)** A sketch illustrating the coupling method using Bio-orthogonal Cu-free click chemistry (dibenzocyclooctyne NHS ester DBCO). Briefly, NHS ester reacts with Lys residues on VLPs and incorporates a cyclooctyne moiety which reacts with the azide labeled molecule forming a stable triazole linkage. **(B)** SDS-PAGE stained with coomassie blue showing the coupling efficiency of p33 to Qβ(CpGs)-VLP using SMPH cross-linker or DBCO cross-linker. Lane 1 protein marker, lane 2 Qβ-VLP monomer, lane 3 Qβ(CpGs)-VLP derivatized with SMPH cross-linker, lane 4 Qβ(CpGs)-p33 vaccine using SMPH cross-linker, lane 5 Qβ(CpGs)-VLP derivatized with DBCO cross-linker, lane 6 Qβ(CpGs)-p33 vaccine using DBCO cross-linker. Each extra band in lanes 4 and 6 indicates a peptide binds to a Qβ monomer. **(C)** Densitometric analysis of SDS-PAGE lanes 1, 4, and 6. (1) uncoupled Qβ-VLP, total 180 monomers, (2) Qβ(CpGs)-p33 vaccine using SMPH cross-linker, and (3) Qβ(CpGs)-p33 vaccine using DBCO cross-linker. Notice the percentage of the coupled peptides to Qβ-VLP monomers, total 180 monomers. **(D)** Percentage of CD8^+^ Tetramer^+^ cells (means ± SEM) in the spleen of vaccinated groups: group 1) Qβ(CpGs)-VLPs, group 2) Qβ(CpGs)-p33 vaccine using SMPH cross-linker, and group 3) Qβ(CpGs)-p33 vaccine using DBCO cross-linker. **(E)** Percentage of CD8^+^ IFN-γ^+^ secreting cells (means ± SEM) in the spleen of vaccinated groups. **(F)** CFSE *in vivo* lytic activity in the spleen of vaccinated groups, using the formula 100X(1–CFSE^Hi^ pulsed/CSFE^Low^ un-pulsed). Statistical analysis by Students *t*-test. **(G)** Representative FACS plots showing the percentage of CD8^+^ Tetramer^+^ cells in the spleen of vaccinated groups. **(H)** Representative FACS histogram of CFSE *in vivo* lytic activity in the spleen of vaccinated groups. (*n* = 3) mice per group, one representative of 3 similar experiments is shown. ^*^P < 0.05; ^**^P < 0.01; ^***^P < 0.001; ^****^P < 0.0001.

For the development of VLP-based vaccines, we previously used SMPH cross-linker to couple the peptide of interest to VLPs (21, 26–28). Linking of cysteine on peptides to lysine on VLPs by SMPH has indeed proven reliable for development of VLP-basedvaccines. However, for the generation of a personalized cancer vaccine, the method may have several disadvantages, namely that epitope internal cysteine will also react with SMPH on VLPs, causing their inactivation and non-reacted SMPH on VLPs is potentially toxic, requiring complex inactivation, and purification procedures after coupling. Therefore, this technique is not well-suited for rapid personalized vaccine production. For these reasons, we established a coupling method based on bio-orthogonal Cu-free click chemistry using a DBCO cross-linker (Figure 2A). This method is non-toxic and thus enables rapid and efficient coupling of regulatory compliant GMP produced VLPs and peptides that can potentially be done at patients' bedside. We compared the efficiency of DBCO based Cu-free click chemistry to the standard SMPH-based method by coupling p33 peptide -as a model antigen- derived from LCMV to Qβ-VLPs loaded with B-type CpGs. Cu-free click chemistry significantly enhanced the coupling of the model antigen to Qβ-VLPs compared to SMPH when assessed by SDS-PAGE (Figure 2B) and densitometric analysis demonstrated enhanced peptide coupling by DBCO compared to SMPH (Figure 2C). In a next step, we compared the immunogenicity of the vaccines generated by the two coupling methods *in vivo*. WT C57BL/6 mice were vaccinated once subcutaneously (s.c.) with Qβ(CpGs)-p33 vaccine prepared with SMPH or Cu-free click chemistry method. Qβ-VLPs were packaged with B-type CpGs for successful activation of DCs and cross-priming. Seven days later, spleens were collected and tested for p33 specific CD8^+^ T-cells by assessing the percentage of p33 tetramer binding and IFN-γ production. Immunization of C57BL/6 mice with the Qβ(CpGs)-p33 vaccine based on Cu-free click chemistry resulted in significant increase in p33 specific CD8^+^ T-cells (*p*. 0.0012) (Figures 2D,G) as well as significant production of IFN-γ (*p*. 0.0421) (Figure 2E) when compared to SMPH method. Lytic activity was also enhanced when assayed *in vivo* by performing CFSE cytotoxicity assay (Figures 2F,H). Thus, Cu-free click chemistry improves vaccine immunogenicity compared to the standard SMPH chemistry.

### Identification and Prediction of CD8^+^ T-Cell Epitopes of B16F10 Melanoma Cells by Immunopeptidomics and Whole Exome Sequencing

**Figure 3:**
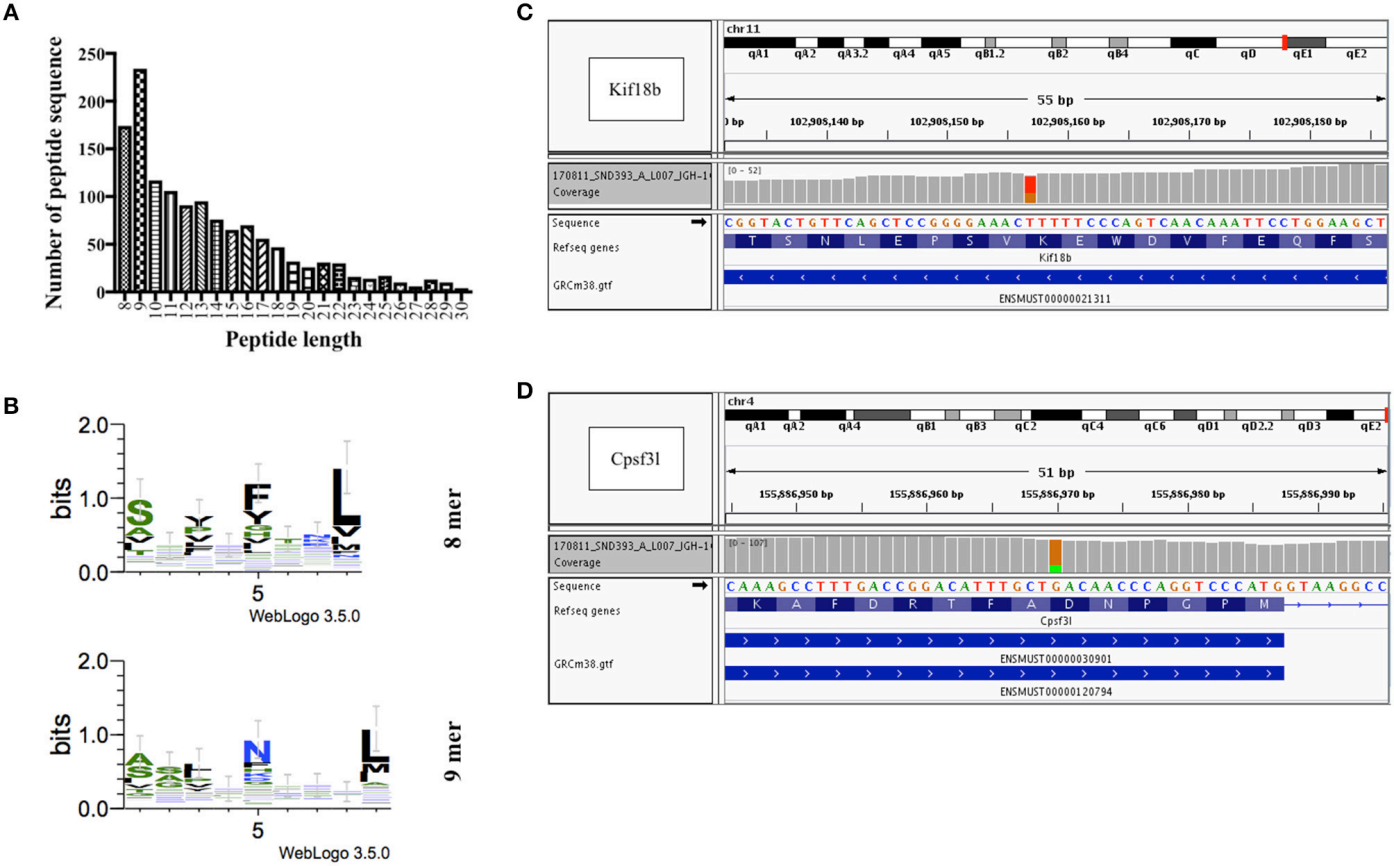
Identification and prediction of CD8^+^ T-cell epitopes of B16F10 melanoma cells by immunopeptidomics and whole exome sequencing. **(A)** Length distribution of peptides identified by immunopeptidomics. **(B)** 8 and 9 mers motifs identified by immunopeptidomics. **(C)** Whole exome sequencing results showing a heterozygous SNV (marked in red and orange) in the 13th exon of gene Kif18b at position Chr11:102,908,157 (ENSMUST00000021311: c.2367T>G, p.Lys739Asn), causing a K>N missense mutation. **(D)** Whole exome sequencing results showing a heterozygous SNV (marked in orange and green) in the 9th exon of gene Cpsf3l at position Chr4:155,886,970 (ENSMUST00000120794: c.1021G>A, p.Asp292Asn and ENSMUST00000030901: c.1087G>A; p.Asp314Asn), causing a D>N missense mutation.

In a next step, we performed immunopeptidomics for B16F10 murine melanoma cell line to identify peptides naturally presented on MHC-I molecules. As expected, a large number of MHC-I-associated peptides was identified (Figures 3A,B). Next, the peptides were evaluated for a number of essential physical parameters including the length, MHC haplotype (H2-D^b^ and/or H2-K^b^) and affinity to MHC-I (high affinity ranking peptides) as summarized in Table 2. Using literature-based assessment of key characteristics such as being melanocyte-specific or a candidate oncogene were also considered. Six peptides were selected from immunopeptidomics for the generation of GL-MTV includes PMEL17, MTC-1, Calpastatin, ZFP518, TRP-2, and Caveoline2.

**Table 2:** The selected peptides from immunopeptidomics for the generation of germline-MTV.

**Peptide**	**Sequence**	**# of a.a**.	**MHC haplotype**	**MHC-I affinity**
Melanocyte protein PMEL (Melanocyte protein Pmel 17) (Premelanosome protein) (Silver locus protein) [Cleaved into: M-alpha; M-beta]	VLYRYGSF	8	H2-K^b^	0.08
MTC-1 Malignant T- cell-amplified sequence 1 (MCT-1) (Multiple copies T- cell malignancies 1)	IGIENIHYL	9	H2-D^b^	0.01
Calpastatin (Calpain inhibitor)	SSPANISSL	9	H2-D^b^, H2-K^b^	0.01
Zinc finger protein 518B	SSVQNKEYL	9	H2-K^b^	0.01
L-dopachrome tautomerase (DCT) (DT) (EC 5.3.3.12) (L-dopachrome Delta-isomerase) (SLATY locus protein) (Tyrosinase-related protein 2) (TRP-2) (TRP2)	SQVMNLHNL	9	H2-K^b^	0.015
Caveolin2	VMYKFLTV	8	H2-D^b^, H2-K^b^	0.015

As for mutated peptides, Castle et al. have previously exploited the mutanome of B16F10 melanoma cell line and identified several non-synonymous point mutations and tested >50 peptides *in vitro* (8). The most promising peptides with mutations in Kif18b and Cpsf3l genes were tested separately *in vivo* for their anti-tumor effect. They used long synthetic peptides (27 a.a.) targeting both CD8^+^ and CD4^+^ cells. Based on these findings we have carried out whole exome sequencing to confirm somatic point mutations in these two specific genes in our B16F10 cell line. We identified several non-synonymous single nucleotide variants (SNVs), including a point-mutation in the 13th exon of the Kif18b gene affecting the transcript ENSMUST00000021311 (Chr11:102,908,157; c.2367T>G; p.Lys739Asn) (Figure 3C), and in the 9th exon of the Cpsf3l gene affecting both ENSMUST00000120794 (Chr4:155,886,970; c.1021G>A; p.Asp292Asn) and ENSMUST00000030901 (Chr4:155,886,970; c.1087G>A; p.Asp314Asn) isoforms (Figure 3D). Accordingly, mutated Kif18b and Cpsf3l peptides were selected for the generation of (Mutated-MTV). We have predicted the affinity of the selected two mutated peptides to H2-D^b^ and H2-K^b^ alleles *in silico* as summarized in Table 3. Please note that we have only selected the best two mutated peptides from B16F10 melanoma model as the mutation burden in most of other cancers is low and therefore it is not always feasible to predict mutated peptides.

**Table 3:** The selected peptides from whole-exome sequencing for the generation of mutated-MTV.

**Peptide**	**Sequence**	**# of a.a**.	**MHC haplotype**	**MHC-I affinity**
Cpsf3l	TFADNPGPM	9	H_2_D^b^	0.8855
Kif18b	FQEFVDWENV	10	H_2_D^b^	1.7

### *In vitro* Validation of Germline and Mutated Epitopes Identified by Immunopeptidomics and Predicted by Whole Exome Sequencing

**Figure 4:**
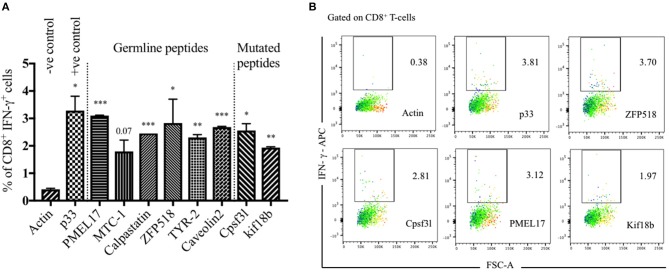
Validation of germline and mutated epitopes identified by immunopeptidomics and predicted by whole exome sequencing. **(A)** Percentage of CD8^+^ IFN-γ^+^ secreting cells (means ± SEM) in duplicate, pre-gated on TILs. p33 peptide was used as a positive control and actin as a negative control. Statistical analysis by Student's *t*-test in comparison with Actin (–ve control). **(B)** Representative FACS plots showing the percentage of CD8^+^ IFN-γ^+^ secreting cells pre-gated on TILs and stimulated with Actin (–ve control), p33 (+ve control), ZFP518, Cpsf3l, PMEL17, and Kif18b peptides. One representative of 3 similar experiments is shown. ^*^P < 0.05; ^**^P < 0.01; ^***^P < 0.001; ^****^P < 0.0001.

Tumor-infiltrating lymphocytes are highly enriched with tumor specific T-cells and thus are the most relevant T-cell population in this regard (29). Therefore, we have used TILs to validate the natural immunogenicity of the six selected peptides from immunopeptidomics and the two mutated epitopes predicted by whole exome sequencing. The selected peptides were synthesized for this experiment as 8 or 9 a.a. to study their recognition by TILs. To this end, TILs were isolated from WT C57BL/6 bearing B16F10 melanoma tumors and cultivated *ex vivo* with IL-2 for 2–3 days. The used melanoma cell line has also been transfected with the model peptide p33 derived from LCMV which was used as a positive control. The activated TILs were then co-cultured with BMDCs pulsed separately with the selected peptides. P33 peptide was used as a positive internal control while actin peptide was used as a negative control due to expected strong immune tolerance. IFN-γ production was then assessed by performing intra-cellular cytokine staining (Figures 4A,B). The results indicated that the selected peptides were effective at inducing significant IFN-γ production by T-cells when compared to actin. There was no significant difference between p33 and the selected germline or mutated peptides.

### Multi-Target VLP-Based Vaccine Using Cu-Free Click Chemistry Could Significantly Hinder the Progression of the Aggressive Transplanted B16F10 Tumor

**Figure 5:**
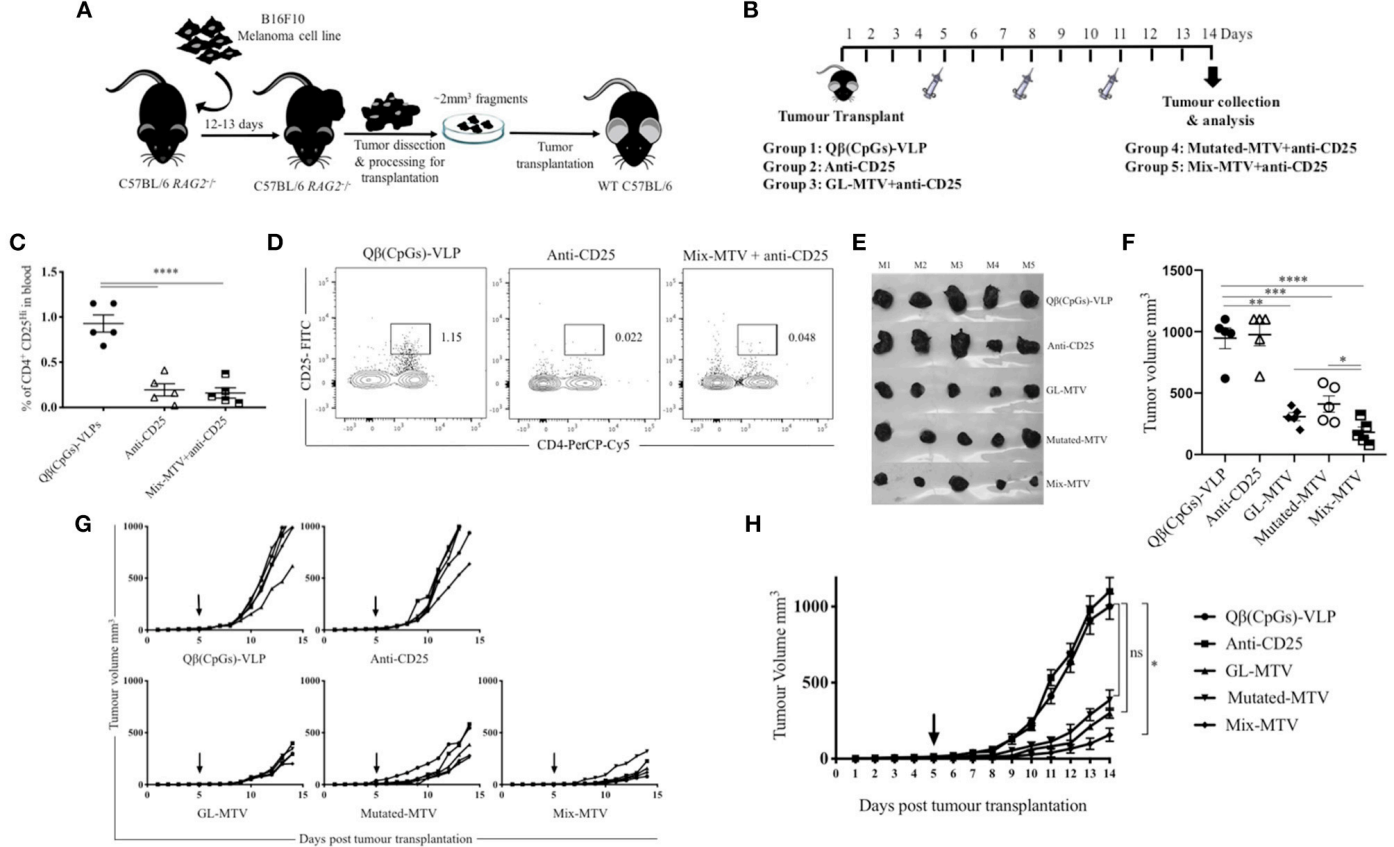
Multi-target VLP-based vaccine using Cu-free click chemistry could significantly hinder the progression of the aggressive transplanted B16F10 tumor. **(A)** A sketch of the challenging therapeutic murine melanoma model based on injecting ~1 × 10^6^ B16F10 melanoma cell line into the flank of C57BL/6 *RAG2*^−/−^ mice. Twelve to thirteen days later the growing tumors are collected and processed for transplantation of ~2 mm^3^ into the flank of C57BL/6 WT mice. **(B)** Vaccination scheme of five groups: Qβ(CpGs)-VLPs, anti-CD25, germline-multi target vaccine (GL-MTV), Mutated-MTV, and Mix-MTV GL-MTV. **(C)** Representative percentage of CD4^+^ CD25^Hi^ in the periphery of the groups vaccinated with Qβ(CpGs)-VLPs, anti-CD25, and Mix-MTV+anti-CD25 on day 6 post-tumor transplantation. **(D)** Representative FACS plots showing the percentage of CD4^+^ CD25^Hi^ in periphery of the groups vaccinated with Qβ(CpGs)-VLPs, anti-CD25, and Mix-MTV+anti-CD25 on day 6 post-tumor transplantation. **(E)** Photographic images of s.c. B16F10 tumors on day 14 post-tumor transplantation. **(F)** Tumor volume mm^3^ (means ± SEM) measured on day 14 post-tumor transplantation for the groups vaccinated with Qβ(CpGs)-VLPs, anti-CD25, GL-MTV, Mutated-MTV and Mix-MTV, each dot represents a tumor. Statistical analysis by Student's *t*-test. **(G)** Individual tumor growth curves of s.c. B16F10 melanoma of the designated groups. **(H)** Combined tumor growth curves of the designated groups, arrows indicate start of treatment. Statistical analysis by AUC. The groups of GL-MTV, Mutated-MTV, and Mix-MTL were combined with anti-CD25mAb in all experiments. (*n* = *5*) mice per group, one representative of 3 similar experiments is shown. ^*^P < 0.05; ^**^P < 0.01; ^***^P < 0.001; ^****^P < 0.0001.

Displaying peptides on CpG-loaded VLPs represents a unique possibility to increase their immunogenicity for T-cells. Hence, Qβ-VLPs were used as a scaffold and packaged with B-type CpGs. The packaging was confirmed by agarose gel and the selected peptides were coupled to Qβ-VLPs using Cu-free click chemistry. The vaccines were produced with germline epitopes (GL-MTV) identified by immunopeptidomics, mutated epitopes (Mutated-MTV) predicted by whole exome sequencing or a combination of both (Mix-MTV). The aim of preparing these groups separately was to compare their overall immunogenicity. The developed vaccines were tested in WT C57BL/6 mice bearing the aggressive B16F10 melanoma tumors. We have adapted a challenging therapeutic murine melanoma model by transplanting ~2 mm^3^ sized B16F10 tumor fragment into the flank of WT C57BL/6 mice and let it grow for 5 more days *in vivo* before vaccination started “Figure 5A.” In order to enhance the efficacy of the developed MTVs, they were combined with anti-CD25 mAb to deplete Tregs and facilitate CTLs infiltration. Anti-CD25 was chosen based on a number of preliminary data showing that anti PD-1 did not enhance the efficacy or immunogenicity of the developed multi-target VLP-based vaccines against B16F10 melanoma cell line, while anti-CD25 did (please note that Daclizumab, the anti-CD25 antibody used in humans, does not deplete Tregs *in vivo*). The transplanted WT C57BL/6 mice were vaccinated s.c. with the developed MTV in combination with low dose of anti-CD25, 3 times over 14 days as the control groups reached the ethical end point by day 14 (Figure 5B). The depletion of Tregs characterized by CD4^+^, CD25^hi^ was assessed in the periphery of vaccinated mice on day 6 post tumor transplantation (Figures 5C,D). All three multi-target VLP-based vaccines could significantly hinder the progression of B16F10 melanoma tumor when assessing the tumor volume at day 14 in comparison to the groups vaccinated with Qβ(CpGs)-VLP or anti-CD25 alone. However, anti-tumor protection was more efficient when vaccinating with Mix-MTV than with GL-MTV or Mutated-MTV (Figures 5E,F). Furthermore, when assessing the growth curve of the aggressive B16F10 tumors over the 14 days, only Mix-MTV showed significant reduction in the tumor growth (*p* = 0.03). Treatment with anti- Qβ(CpGs)-VLP or CD25 mAb alone did not show any anti-tumor protection (Figures 5G,H).

### Mix Multi-Target VLP-Based Vaccine Increased CD8^+^ T-Cell Infiltration Into B16F10 Tumor and Enhanced the Survival

**Figure 6:**
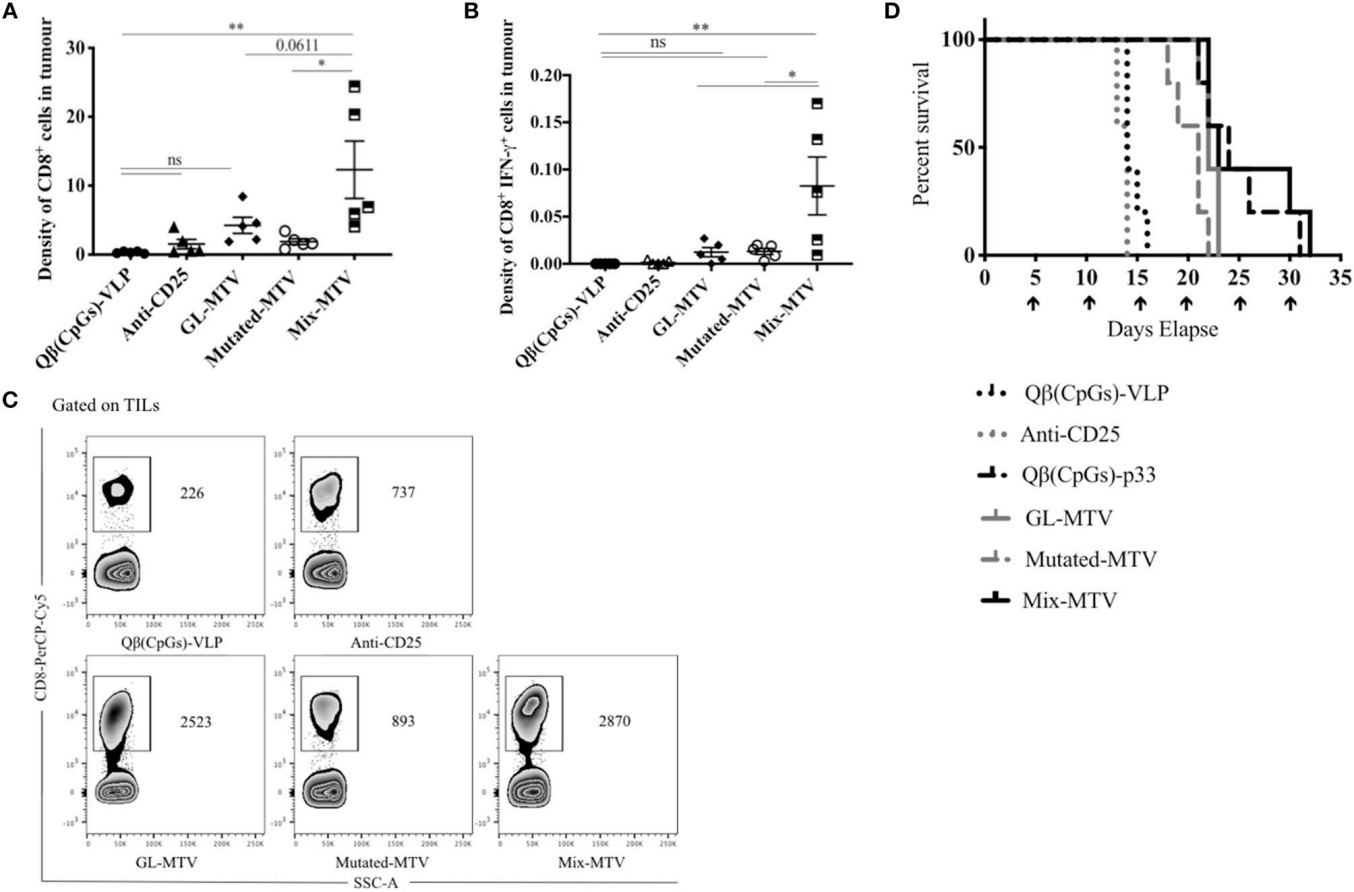
Mix multi-target VLP-based vaccine increased CD8^+^ T-cell infiltration into B16F10 tumor and enhanced the survival. **(A)** Density of CD8^+^ T-cells (means ± SEM) in tumor, pre-gated on TILs in the groups vaccinated with Qβ(CpGs)-VLPs, anti-CD25, GL-MTV, Mutated-MTV, and Mix-MTV. The density measured by dividing the total number of CD8^+^ T-cells in each tumor by its volume. **(B)** Density of CD8^+^ IFN-γ^+^ secreting cells (means ± SEM) in tumor, pre-gated on TILs in the vaccinated groups. Statistical analysis by one-way ANOVA. **(C)** Representative FACS plots showing the total number of CD8^+^ T-cells in each tumor in the vaccinated groups. **(D)** Survival of mice in the vaccinated groups, mice were euthanized when the tumor reached 1,000 mm^3^. The arrows indicate vaccination time. Statistical analysis by log-rank test. The groups of GL-MTV, Mutated-MTV, and Mix-MTL were combined with anti-CD25mAb in all experiments. (*n* = *5*) mice per group, one representative of 3 similar experiments is shown. ^*^P < 0.05; ^**^P < 0.01; ^***^P < 0.001; ^****^P < 0.0001.

It is readily accepted that high-grade CD8^+^ T-cell density in tumors is correlated with better prognosis and is an essential piece of evidence for effective immune responses (7, 30). Based on that, we measured the total number of infiltrating CD8^+^ T-cells in the tumors and calculated their density in each vaccinated group (by dividing the total number of CD8^+^ T-cells in each tumor by its volume). Mix-MTV significantly increased CD8^+^ T-cells density when compared to the groups vaccinated with Qβ(CpGs)-VLPs (*p*. 0.0028), anti-CD25 (*p*. 0.0076), or Mutated-MTV (*p*. 0.01). The statistical difference between Mix-MTV and GL-MTV was borderline significant (*p*. 0.06) (Figures 6A,C). When assessing the density of CD8^+^ IFN-γ^+^ secreting cells in the tumors, the data showed that Mix-MTV group induced the strongest cytokine production which was superior to the groups vaccinated with GL-MTV or Mutated-MTV (Figure 6B). These results underline the effectiveness of combining both germline and mutated epitopes in one vaccine when treating the aggressively growing B16F10 melanoma tumor.

Whether the different multi-target VLP-based vaccines can extend the life-span of tumor-bearing mice was tested next. Tumors in control mice or mice treated with anti-CD25 mAb alone reached their ethically allowed maximal size of ~1,000 mm^3^ within 13–16 days, underscoring the aggressiveness of the model used. Vaccination with the GL-MTV or Mutated-MTV in combination with anti-CD25 mAb extended the mouse life-span by about 8 days while the Mix-MTV did so by about 16 days (Figure 6D). In fact, vaccination with the Mix-MTV reached protective levels of the vaccine based on p33 peptide, one of the strongest T-cell epitopes known in mice.

### Mix Multi-Target Vaccine Altered the Myeloid Composition of B16F10 Tumor

**Figure 7:**
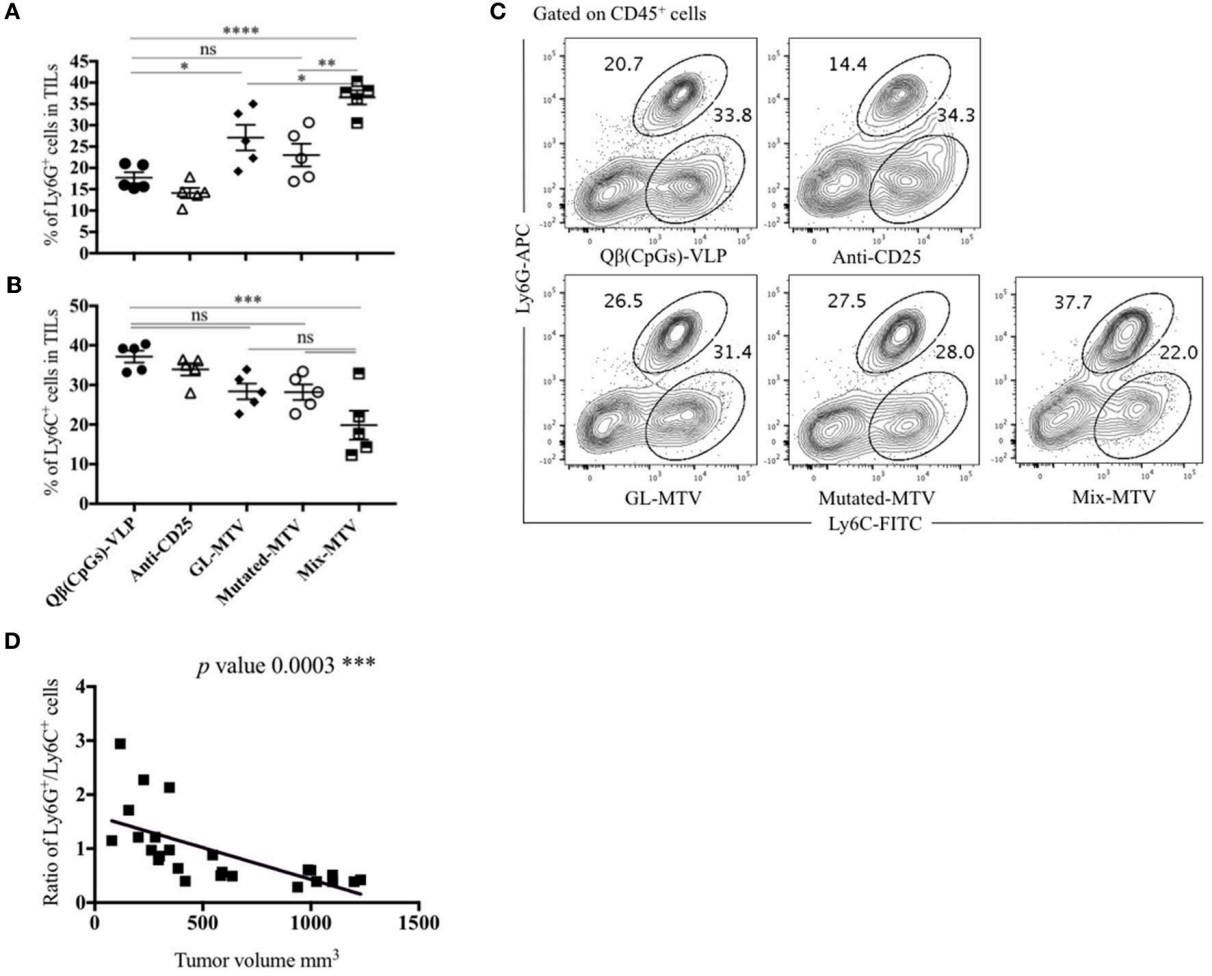
Mix multi-target vaccine altered the myeloid composition of B16F10 tumor. **(A)** Percentage of Ly6G^+^ cells (means ± SEM) in TILs, pre-gated on CD45^+^ cells in the groups vaccinated with Qβ(CpGs)-VLPs, anti-CD25, GL-MTV, Mutated-MTV, and Mix-MTV. **(B)** Percentage of Ly6C^+^ cells (means ± SEM) in TILs, pre-gated on CD45^+^ cells in the vaccinated groups. Statistical analysis by one-way ANOVA. **(C)** Representative FACS plots showing the percentage of Ly6G^+^ and Ly6C^+^ cells in TILs in the vaccinated groups. **(D)** Correlation between the ratio of the percentage of Ly6G^+^ and Ly6C^+^ cells over the tumor volume mm^3^ in the vaccinated groups. Statistical analysis by linear regression. The groups of GL-MTV, Mutated-MTV, and Mix-MTL were combined with anti-CD25mAb in all experiments. (*n* = *5)* mice per group, one representative of 3 similar experiments is shown. ^*^P < 0.05; ^**^P < 0.01; ^***^P < 0.001; ^****^P < 0.0001.

We have studied next the effect of the vaccination with the prepared multi-target vaccines on tumor myeloid immune cell composition. Specifically, we have looked at both granulocytic and monocytic myeloid populations in TILs. There was an overall increase in the Ly6G^+^ granulocytic population in the groups vaccinated with Mix-MTV and GL-MTV in comparison to the control groups Qβ(CpGs)-VLPs or anti-CD25. However, the infiltration of Ly6G^+^ granulocytes into the tumour was significantly higher in the group vaccinated with Mix-MTV when compared to the groups vaccinated with GL-MTV (*p*. 0.0331) or Mutated-MTV (*p*. 0.0015) (Figures 7A,C). The increase in the granulocyte population was accompanied by a decrease in monocytic population characterized by Ly6C^+^ which is shown prominently in the group vaccinated with Mix-MTV (Figures 7B,C). The observed increase in granulocytic population and the decrease in monocytic one was inversely correlated with the tumor volume (*p* 0.0003) as shown in (Figure 7D) and is compatible with induction of a more protective environment (31, 32).

## Discussion

VLPs have shown to be a promising platform for the development of effective vaccines. Such platforms significantly enhance the immunogenicity of tumor epitopes and help overcoming tolerance and anti-inflammatory milieus (33, 34). Indeed, it has been shown previously that a VLP-based vaccine can be utilized for active immunization against melanoma and vaccination against a single epitope resulted in strong CTL responses and exerted therapeutic pressure to the extent of provoking outgrowth of antigen escape variants in mice and humans (17, 18, 35). Personalized cancer vaccines targeting the patient's tumor specific mutanome may have the potential to generate clinically effective T-cell responses (14, 15). In previous studies, multi-target long peptides, or RNA-based vaccine have been used to induce CD8^+^ (and CD4^+^) T-cell responses in melanoma patients. Interestingly, even though the vaccines were designed to induce CTL responses, they preferentially stimulated CD4^+^ T-cells, reminiscent of the generally acknowledged difficulties in mobilizing strong and broad CD8^+^ T-cell responses by vaccination (14, 15, 36, 37). Nevertheless, these findings support the concept that a multi-target vaccine covering a broader range of cancer antigens can control tumor progression and avoid outgrowth of antigen-escape variants.

In this study, a novel platform for the generation of a personalized VLP-based vaccine has been developed by combining both immunopeptidomics and whole exome sequencing techniques. Earlier versions of VLP-based vaccines using SMPH chemistry to couple antigens of interest to VLPs showed good efficacy. SMPH reacts with free Lysine on the VLP and free cysteine on the peptides. However, not only cysteines of the linker but also cysteines within the target epitope may react with SMPH, resulting in the inactivation of cysteine-containing epitopes. In addition, non-reacted SMPH on VLPs may be toxic as it can react with cysteines in the body after injection of the vaccine. To overcome this problem and enable mixing and coupling of peptides with VLPs at bedside, a new coupling chemistry should be employed that leaves cysteines untouched and that will not react with anything in the host. To this end, bio-orthogonal Cu-free click chemistry has been implemented here for GMP-compatible production at bedside. The results show that DBCO is superior to SMPH cross-linker in terms of (1) coupling efficiency to Qβ-VLPs and (2) in inducing CTL and IFN-γ response *in-vivo*. Bio-orthogonal in general, refers to any chemical reaction that can occur in the living organism without causing cellular toxicity or interfering with natural biological reactions (38). Furthermore, the azide groups attached to the peptides are metabolically stable and lack any reactivity with natural biological functionalities in the cells as it only reacts with the alkyne groups (39, 40). This new coupling method has shown high selectivity for *in vivo* studies (41) such as for labeling, imaging, and tracking cells (39, 42). Cu-free click chemistry has been recently applied for the synthesis of glucoconjugate O-antigen vaccine coupled to CRM_197_ carrier protein against Salmonella Typhimurium (43).

Identification of optimal cancer-specific antigens remains a priority in the field of cancer immunotherapy. Some scientists favor peptides that are actually presented on the tumor cells using immunopeptidomics and others rely on epitope prediction based on whole exome sequencing. The promising clinical trials conducted so far to test a personalized cancer vaccine have mainly used whole exome sequencing validated by RNA-seq to predict tumor-specific neoantigens. Immunopeptidomics identifies peptides that are actually presented but requires relatively large amounts of tumor-tissue. By comparison, whole exome sequencing can be performed with minimal cell numbers, but epitopes are only predicted and their presence is not actually physically assessed. In addition, the relative merits of germ-line vs. mutated epitopes for vaccine design is also disputed. While the former may be conserved in many patients, the latter likely induce less or even no immune tolerance, as they are not expressed in the thymus during early development nor in the periphery other than in tumor cells (14, 15, 44). In our study, to directly compare the different approaches, both immunopeptidomics and whole exome sequencing were performed to identify and predict both types of T-cell epitopes. Several T-cell epitopes have been identified and prioritized by bioinformatics, as well as by their ability to stimulate TILs from tumor-bearing mice. TILs are highly enriched with specific T-cells and thus the most relevant T-cell population for anti-tumor T-cell responses. Using these approaches, two sets of peptides, germline and mutated epitopes, were identified and displayed on VLPs loaded with CpGs. In recent years, it has become evident that anti-tumor response may be enhanced in the presence of checkpoint inhibitors. Blocking PD-1 vs. depleting Tregs using anti-CD25 mAb was compared. While blocking PD-1 had minimal impact on B16F10 tumor growth, anti-CD25 mAb substantially enhanced protection. Therefore, the developed multi-target VLP-based vaccines were combined with anti-CD25 mAb treatment. The results showed that using germline peptides or mutated or a mixture of both can confer protection against tumor growth in B16F10 melanoma model. Interestingly, mixing both types of epitopes was most promising. This effect was also shown when calculating the density of CD8^+^ T-cells or CD8^+^ IFN-γ^+^ secreting cells in TILs and when assessing the effect of the vaccines on the myeloid composition of the tumor. Indeed, protection using the Mix-MTV was as potent as observed with the artificial model antigen, peptide p33, one of the strongest T-cell epitopes known in mice.

It has been shown previously that eliminating large established tumors is challenging even in pre-clinical studies. Kelly et al. has shown that curing a B16F10 melanoma tumor sized 60–80 mm^2^ is possible when combining four immunotherapeutic agents (31). Here, we have used a more challenging model employing transplanted solid tumors rather than single cell suspension, resulting in tumors with physiological stroma and vascularization. Combining the multi-target VLP-based vaccine with anti-CD25 doubled the lifespan of the transplanted mice and changed the immunological milieu of the tumors, which is an impressive result given the model used.

The approach of vaccinating with only two neoantigens and combining them with germ-line antigens avoids the need for identifying large numbers of neoantigens that may represent a particular problem in tumors of low mutational burden. A prominent example of this is the aggressive metastatic breast cancer murine cell line 4T1. It has been shown that 4T1 harbored 27 mutations in their expressed genes, however only one mutation was predicted to bind MHC-I H2-D^b^ allele (45). These data are consistent with the fact that human tumors associated with environmental mutagens such as melanoma have more mutations than other cancers (46). Additionally, Keeping the numbers of neoantigens low may also simplify the production of this type of personalized vaccines to some extent, as the final vaccine formulations would vary less from one patient to the next one, easing the regulatory challenges for producing and releasing these pharmaceutical products for clinical use.

In conclusion, this study proposes a novel platform for the development of an immunogenic personalized melanoma vaccine based on VLPs. This vaccine utilizes CTL epitopes predicted and validated by both immunopeptidomics and whole exome sequencing techniques. The peptides were coupled to the highly immunogenic VLPs by Cu-free click chemistry, representing a fast, safe and efficient method of coupling, enabling GMP compliant production for future use in clinical settings. The platform developed here can be used to target potentially any malignant tumor and thus can be applied broadly in cancer medicine.

## Ethics Statement

Animal procedures were conducted at the University of Bern, Switzerland, in accordance with the Swiss Animals Act (455.109.1, September 2008, 5th, University of Bern).

## Author Contributions

MM, MV, CR, DS, and MB: design of experiments, acquisition of data, interpretation of data, analysis of data. NT and JM: immunopeptidomics acquisition and data analysis. JM and SS: exome sequencing data analysis. MM, MV, AE-T, GC-M, AG, CR, TK, AK, DS, SD, and MB: writing, revise and revision of manuscript. MM, MV, and CR: technical and material support. MB: study supervision.

### Conflict of Interest Statement

The authors declare that the research was conducted in the absence of any commercial or financial relationships that could be construed as a potential conflict of interest.
